# Study protocol for a randomised controlled trial of a cognitive-behavioural prevention programme for the children of parents with depression: the PRODO trial

**DOI:** 10.1186/s12888-014-0263-2

**Published:** 2014-09-18

**Authors:** Belinda Platt, Kathrin Pietsch, Kathrin Krick, Frans Oort, Gerd Schulte-Körne

**Affiliations:** Department of Child and Adolescent Psychiatry, Psychosomatics and Psychotherapy, Ludwig-Maximilians-University, Nußbaumstraße 5a, 80336 Munich, Germany; Research Institute of Child Development and Education, Faculty of Social and Behavioural Sciences, University of Amsterdam, Nieuwe Achtergracht 127, 1018 WS Amsterdam, Netherlands

**Keywords:** Paediatric, Adolescent, Psychiatry, Cognitive behavioural therapy, Parental depression, RCT, Offspring, SPIRIT, Preventive

## Abstract

**Background:**

Depression is one of the most common psychiatric illnesses worldwide, but is nevertheless preventable. Since the children of parents who have depression are at greatest risk of developing depression themselves, prevention programmes for this population are a major public health priority. Here we report the study protocol of a randomised controlled trial of a group-based psychological intervention for families with i) at least one parent who suffers (or has suffered) from depression and ii) at least one child who has no current or previous psychiatric diagnosis.

**Methods/Design:**

Eligible families will be randomly allocated to receive either a German adaptation of the 12-session cognitive-behavioural Raising Healthy Children intervention (Gesund und glücklich aufwachsen; N = 50), or no intervention (usual care; N = 50). The primary outcome (child diagnosis of an episode of depression) will be assessed at 15-month follow-up. The secondary outcomes (child psychopathological symptoms) will be assessed immediately following completion of the intervention (6-months), as well as at 9- and 15-month follow-up. We hypothesise that children in the intervention condition, compared with those who do not receive the intervention, will show fewer symptoms of psychopathology, and be less likely to meet diagnostic criteria for a depressive episode, at follow-up.

**Discussion:**

Despite their elevated risk of developing depression, there is little formal support available for the children of parents with depression. This study provides an important step in the development of more effective depression prevention measures, which are needed if the personal, social and economic burden of depression is to be reduced.

**Trial registration:**

Clinical Trials NCT02115880. Registered April 7 2014.

## Background

Depression is one of the most common psychiatric illnesses worldwide, with lifetime prevalence rates of an episode of major depressive disorder ranging from 8-12% globally [1]. World Health Organisation predictions suggest that by 2020, depression will be a major cause of disability worldwide, second only to ischaemic heart disease [2]. Thankfully, selective and indicated prevention programmes, which target populations at elevated risk of depression, suggest that depression is preventable [3]. One of the groups at greatest risk of experiencing an episode of depression is the children of parents who suffer, or have suffered, from depression [4]. Adolescent depression is associated with negative educational and social outcomes [5,6], suicidality [7], and psychiatric problems in adulthood [8]. Developing selective preventive interventions that reduce the risk of depression for the children of parents with depression is therefore a major public health priority [9]. Here we describe a randomised controlled trial (RCT) of a selective prevention intervention for children and adolescents with a parent who has or is suffering from depression.

For the children of parents with depression, the risk of developing depression by the age of 20 may be as high as fifty percent [10]. A prospective cohort study which followed 182 adolescents of depressed and non-depressed parents into adulthood, found that ten years after being initially assessed, individuals whose parents had depression at assessment were four times as likely to have developed depression themselves, compared to those without a parent suffering from depression [11]. Twenty years on, when participants were in their mid-thirties, the risk remained around three times as high [12]. Across the whole study period, the most common period for the onset of a depressive episode was between the ages of 15 and 20 years. The mechanisms by which the risk of depression is transferred from parent to child are thought to be a complex interaction of biological, social, and psychological processes, but nevertheless amenable to change [13]. For example, depressive symptoms such as irritability, sadness and social withdrawal [14] often elicit maladaptive parenting behaviours, such as unresponsiveness or over-intrusion, which by causing the child distress, are thought to be associated with poorer child emotional development [9]. Children of parents with depression may also learn maladaptive coping strategies such as attributing failure to personal, inherent characteristics, and ruminating over difficulties, which further exacerbate the ways in which they deal with the stress associated with their parents’ depression [15]. These factors have, to varying degrees, formed the basis for existing preventive interventions for the children of parents with depression [9,16-20].

The Family Talk Intervention (FTI), developed by Beardslee and colleagues in the USA [20-22], is a *family-based* programme which provides *psycho-education* to affected families in an attempt to raise awareness of the negative impact of parental depression on child emotional development. Although this intervention appears to improve families’ knowledge of depression and communication skills [21,22], it places little emphasis on using psychological strategies to modifying negative cognitions and coping behaviours. Furthermore, there is no evidence that it is more effective in reducing internalising symptoms than a simple non-clinician-delivered education condition [22]. In contrast, the Coping With Depression (CWD) programme developed by Clarke and colleagues [19], also in the USA, aims to directly modify coping strategies through structured *cognitive-behavioural therapy* (CBT) delivered to *children alone*. The first trial of the intervention effectively reduced child depression diagnoses at 12-month follow-up [19]. These findings have since been replicated in a large-scale RCT of a modified version of the CWD, in which children in the experimental group showed significantly fewer episodes of depression than those in a usual care condition at 9-month (21.4% vs. 32.7%) [17] and 24-month follow-up (36.8% vs. 47.7%) [16] respectively. Although the findings are promising, this study also included children who had previously had an episode of depression themselves. Since recurrent depression is a predictor of poorer treatment outcomes, even better response rates might be achieved by targeting adolescents who have never experienced an episode of depression [16]. Given that such programmes are founded on the premise that parental depression plays an important role in predicting child outcomes, involving parents is likely to be important aspect of a successful preventive intervention [9,23,24].

A third preventive intervention, Raising Healthy Children (RHC), has combined these two previous approaches and by administering CBT to children, and parenting skills training to affected parents [9,25]. At 12-month follow-up the rate of depression in children in the family-based intervention group was significantly lower (8.9%) than that of a self-study control group (20.8%) [9]. These positive effects remained at 24-month follow-up [25] but are yet to be replicated.

More generally, prevention programmes for the children of depressed parents have largely been developed and evaluated in the USA [9,17,19,20], with the exception of a study which evaluated the FTI in Finland [18,26]. One quasi-experimental study conducted in Germany compared the effects of a family-based intervention for the children of parents with a psychiatric disorder (not specifically depression) but only measured effects on life-quality and social support (not symptoms or a diagnosis of depression) [27]. Another quasi-experimental study of a selective prevention programme in Germany (EFFEKT-E) administered an intervention to children aged 4–7 whose mothers (but not fathers) had depression [28].

Here we describe the study protocol (Version 1; February 2014) of an RCT comparing a German-language adaptation of the RHC prevention programme with usual care (no routine intervention) for psychiatrically healthy children with a parent who is depressed. To our knowledge, this is the first RCT of its kind to be conducted in Germany. Given previous findings which suggest that interventions may be more effective when parents are not currently depressed [17], we also include non-depressed parents who have experienced a depressive episode during the child’s lifetime. Although we will include children showing sub-clinical symptoms of depression, they will be excluded if they meet diagnostic criteria for a current or previous episode of a psychiatric disorder, including depression. Our hypothesis is that in comparison to a control group who receive no intervention, participants in the experimental group will show a reduced risk of depression as indexed by i) fewer cases of a depressive episode at 15-month follow-up, and ii) fewer psychopathological symptoms at 9- and 15-month follow-up. We expect these improvements to be associated with improvements in the child’s knowledge of depression, the child’s coping strategies and attributional style, and the parents’ parenting skills. Based on previous studies [9,16,17], we expect children whose parents who are not depressed at baseline to show the greatest benefits from the programme. Since the programme is designed to reduce depression by modifying children’s responses to stress, we expect families who experience more stressful life events during the course of the study to show greater benefit from the intervention than those who experience less.

## Methods/Design

The study design is reported in line with the SPIRIT 2013 Statement (Standard Protocol Items: Recommendations for Interventional Trials) [29]. The study has received ethical approval from the Ludwig-Maximilians-University (LMU) Medical Division Ethics Committee, Munich, Germany (Study ID: 3–14).

### Design

**Figure 1:**
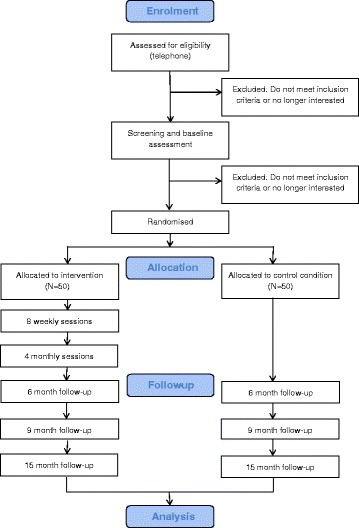
**Overview of the Prodo study design.**

Figure 1 provides an overview of the study design. The study is a randomised controlled trial (RCT) of a preventive intervention to reduce the risk of depression for 100 psychiatrically healthy children with a parent who suffers from depression. In a parallel assignment design, the study will compare the effectiveness a 6-month family- and group-based cognitive-behavioural intervention (N = 50) with no intervention (usual care; N = 50). Following an initial assessment session (T1), families will be randomised to one of the two groups.

Randomisation will be performed by a statistician (FO) in blocks of eight families (four per group), stratifying for parental diagnostic status (current or previous depression). Families in both groups will take part in a total of three outcome assessment sessions: (T2) immediately after the intervention (6 months after baseline), (T3) nine months after baseline, and (T4) fifteen months after baseline. The single-blind design will mean that although participants are aware of the condition they have been assigned to, outcome assessors will not.

### Participants

Eligible families are those in which i) the participating parent(s) fulfils diagnostic criteria (according to the Diagnostic and Statistical Manual for Mental Disorders; DSM-V) for a current episode of depression, or reports an episode of depression during the child’s lifetime which fulfils the same diagnostic criteria, and ii) the participating child is aged 8–17, and has an IQ of at least 85. In line with the procedure of Compas and colleagues [9], all siblings who meet the inclusion criteria can take part in the study and will be allocated to the same group. If both parents fulfil the study inclusion criteria they may both take part. If the partner (or other adult living in the house with parenting responsibilities) of the participating parent does not have a diagnosis of depression, they may still take part in the study, providing they do not meet the exclusion criteria (see below). All study participants must have adequate German-language skills and provide written informed consent to take part in the study and to the video-recording of intervention sessions for the purposes of quality control.

Families will be excluded from the study if i) the participating parent(s) has current symptoms of bipolar disorder, psychosis, personality disorder, substance abuse, is suicidal or in crisis, or has serious symptoms of another disorder that may hamper their ability to take part, and ii) the participating child fulfils diagnostic criteria for a current (or previous) episode of any psychiatric disorder, or is undergoing (or has undergone) treatment for depression, iii) the family takes part in family therapy during the course of the study period. Children will also be excluded if they are in crisis or have serious symptoms of a disorder that may hamper their ability to take part in the study.

### Recruitment

Families will be recruited through multiple sources. Adult patients from psychiatric clinics in Munich who are eligible for the study will be invited by their therapist to take part, given written information, and encouraged to make contact with the study team. Advertisements will also be placed in the general public as well as in general practices in Munich. Parents or children who have been involved in previous studies by the study group and who have declared an interest in taking part in future studies will also be invited to take part in the study with their child. Each family will receive €50 for every child that participates (€25 at T1 and €25 at T4).

### Procedure

When both the child and their parent are interested in taking part and make contact with the study team, they will be informed about the details of the study (and the inclusion/exclusion criteria) over the telephone. If they are suitable and still interested, a separate appointment for a screening session will be made. Here the parent and child will be given an overview of the study (including the fact that their allocation to the experimental/control group will be decided at random) and written informed consent will be taken from both the parent and child. Following a diagnostic (screening) interview, baseline measures of all outcome measures (see below) will be administered. If only one parent attends the screening session, the other parent will nevertheless be asked to complete self-report questionnaires about symptoms of general psychopathology and depression specifically. After the appointment, a decision about the family’s suitability for participation in the study will be made, and if suitable, randomisation will be performed. The screening session will be conducted and analysed by someone trained and experienced in conducting psychiatric assessments but who will not be involved in delivering the intervention to the family. At 6- (T2) and 9-months (T3) after baseline, participants will be asked to complete outcome measure questionnaires by post, telephone or electronically. All participants will be invited back for an appointment at 15-month follow-up where the final outcome assessment (T4) will take place.

### The prevention programme

The prevention programme (Gesund und glücklich aufwachsen; GGA) is an adaptation of the RHC programme developed and evaluated by Compas and colleauges. A full description of the original RHC programme is provided elsewhere [9,25]. The manualised programme is a group-based cognitive-behavioural intervention designed for four families, which includes both affected parents and psychiatrically healthy children. The manual and work materials have been translated into German and adapted to German culture. Intervention sessions will be recorded and later analysed for treatment integrity.

The intervention is composed of a total of eight weekly sessions and four monthly booster sessions, with homework tasks for parents and children given after each session. For the first three sessions, parents and children take part together. The key aim of these group-sessions is psycho-education (improving knowledge about depression). Themes include symptoms of depression to be aware of, the meaning of the illness for the child themselves, and the effect of the parent–child interaction on mental health. Sessions four to eight will be conducted separately for children and parents. In these sessions children learn coping strategies (acceptance, distraction, activities and positive thinking) and parents learn more adaptive parenting skills. The four booster sessions, in which the children and parents are brought back together, are designed to help solve problems that arise, strengthen coping and parenting skills, and give new homework.

The sessions last two hours each and will be delivered by two sufficiently qualified study members (doctoral students, qualified therapists, and trainee clinical psychologists) in the Department of Child and Adolescent Psychiatry, University Hospital Munich. All study members will be trained by a qualified clinical psychologist in how to administer the manual.

### Control group

In contrast to Compas and colleagues [9], who compared the prevention programme to a written information condition, we follow the procedure of Garber and colleagues [17] by not offering any intervention in the control group. The no intervention control condition will enable us to test a true preventive effect of the intervention (see Compas et al. [9] for a discussion of how active-control conditions restrict conclusions about preventive effects), in addition to being able to estimate the clinical value and cost-effectiveness of the intervention in comparison to currently available support. One limitation of usual care control conditions is that they do not enable the intervention’s mechanisms of action to be tested. However, we intend to address this by measuring numerous hypothesised mechanisms of action (knowledge of depression, parenting skills, child’s coping skills, attributional style) in both groups. The complexity of depression prevention interventions (group-based, multiple active components, therapist-lead) means that active-control conditions used in this field (commonly self-study) are neither able to identifying mechanisms of action nor control for placebo effects. Participants allocated to the control condition of our study will be able to take part in the intervention after the study period.

### Measures

Table 1 provides a list of screening, outcome, mediator and moderator measures.

**Table 1:** **Measures to assess screening, outcome, mediator and moderator variables**

**Function**	**Measure**	**Instrument**	**T1**	**T2**	**T3**	**T4**
Screening	Diagnostic status (parent)	DIPS [30]; SCID-II [31]	X			
	Diagnostic status (child)	K-DIPS [34]	X			
	IQ (child)	CFT-20-R [35]	X			
	Symptoms of psychopathology and depression (non-participating parent)	SCL-90R [32]; BDI-II [33]	X			
Outcome	Diagnostic status (child)	K-DIPS [34]				X
	Psychopathological symptoms (child)	DIKJ [36]; BDI-II [33]; YSR [39]; CBCL [38]	X	X	X	X
Confounding variables	Demographics (age, gender, education level, family income, marital status, nationality)	Self-report	X			
	Depression severity (parent)	BDI-II [33]; DIPS [30]	X			X
	Course of therapy (type, duration and location; parent)	Self-report	X			X
Mediators	Knowledge of depression (child)	Allgaier et al. [40]	X	X	X	X
	Coping strategies (child)	FEEL-KJ [41]	X	X	X	X
	Parenting style	ESI [42]	X	X	X	X
	Attributional style (child)	ASF-KJ [43]	X	X	X	X
Moderators	Stressful life events (child)	CASE-C/P [44] (child and parent report)				X
	Parental diagnostic status (current vs. previous)	DIPS [30]	X			

#### Screening measures

The Diagnostic Interview for Psychiatric Disorders (DIPS) [30] will be used to identify whether or not the participating parent(s) fulfils the inclusion criteria of having a current or previous episode of depression (according to DSM-V criteria), and the exclusion criteria of current symptoms of bipolar disorder, psychosis, substance abuse, or suicidality. The DIPS is a clinician-administered semi-structured interview used to ascertain diagnoses in both psychiatric patients and community samples. It enables the researcher to assess both current and retrospective episodes of depression. The Structured Clinical Interview for Axis II DSM Disorders (SCID-II) [31] is a standardised instrument for assessing symptoms of personality disorder, which will be used to assess whether the participating parent fulfils the exclusion criteria of personality disorder. The SCID-II begins with a screening questionnaire, which is followed by a semi-structured interview if the participant indicates symptoms of a personality disorder in the questionnaire. The questionnaires SCL-90R [32] and BDI-II [33] will be administered to non-participating parents to assess symptoms of general psychopathology and depression respectively.

The Diagnostic Interview for Psychiatric Disorders for Children and Adolescents (K-DIPS) [34] will be used to identify whether or not children meet the study exclusion criteria of having a psychiatric diagnosis (current or previous; according to DSM-V criteria). The K-DIPS is designed for children aged 6–18 years old and is a standard instrument used to assess clinical diagnoses in children and adolescents. Both diagnsotic instruments will be delivered by members of the team trained in using the manual.

The German-version of the Culture Fair Intelligence Test (CFT 20-R) [35] will be used to assess whether children fulfil the inclusion criteria of having sufficient cognitive capability to perform the prevention programme (IQ ≥ 85). The CFT 20-R is made up out of two similarly structured test parts, each with four sub-tests (continuous rows, classification, matrices and topological conclusions) and is suitable for children aged 8–19. In this study, only part one of the test will be used. The CFT 20-R has very good test-retest reliability (0.80-0.82) and internal consistency (0.95) and correlates well with other intelligence tests [35].

#### Outcome measures

The primary outcome is the presence or absence of a *depressive disorder* in the child at 15-month follow-up (K-DIPS). The secondary outcome of the study is the severity of *psychopathological symptoms* immediately after the intervention (T2; 6-months), at 9-month follow-up (T3) and 15-month follow-up (T4). Self-reported symptoms of depression will be collected using the Depression Inventory for Children and Adolescents (DIKJ; children aged 8–12) [36] and the German-version of the revised Beck Depression Inventory (BDI-II; children aged 13 and over) [33]. Both measures are established measures of depression symptom severity with very good internal consistency (0.92 [36] and 0.90 [37] respectively) and good correlations with other measures of depression in this age-group. More general internal and external symptoms will be collected using the german versions of the Child Behaviour Checklist (CBCL/4-18 [38]; parent-report) and the Youth Self-Report (YSR/11-18 [39]), both standard measures used in previous studies of child and adolescent depression prevention [9]. The CBCL/4-18 assesses both internalizing and externalizing behaviours using 118 items which pertain to the frequency of a given behaviour (scored on a three-point likert scale). The YSR allows the youth themselves to report on the same behaviours described in the CBCL. The CBCL and YSR both have good test-retest reliability and good to very good internal consistency [38,39].

#### Confounding variables

Age (child), gender (parent and child), education level (parent), family income, marital status, nationality, parental depression severity (BDI-II [33] and DIPS [30]) and parental depression treatment (type, duration and location) will be treated as potentially confounding variables.

#### Mediators

A validated questionnaire developed by our research group [40] will be used to determine whether the programme has improved children’s *knowledge of depression*.

The Questionnaire for the Assessment of Emotion Regulation in Children and Adolescents (FEEL-KJ) [41] will be used to assess the potentially mediating role of *emotion regulation*. The questionnaire identifies adaptive emotion regulation strategies such as problem-oriented approach, distraction, lifting mood, acceptance, forgetting, reappraisal and cognitive problem-solving, as well as maladaptive coping strategies such as giving up, aggressive behaviour, withdrawal, self-deprecation and perseveration. The questionnaire has good internal consistency (0.69-0.91) and test-retest reliability (0.62-0.81).

We will assess the potentially mediating role of *parenting style* using the parenting style inventory (ESI) [42], a two-part child-report questionnaire. The first part contains 60 items which assess support, restraint, praise, criticism, and inconsistency. The second part contains five questions which assess the intensity of punishment. The test shows moderate test-retest reliability (0.51-0.72) and good internal consistency on the two parts respectively (0.77-0.92 and 0.65-0.71).

The Attribution Style Questionnaire for Children and Adolescents (ASF-KJ) [43] is a self-report questionnaire which we will use to assess the mediating role of *attributional style*. Participants are asked to identify the internality, stability, and globality of eight positive and negative events. The internal consistency of the stability and globality constructs lies between 0.72 and 0.81.

#### Moderators

The moderating role of *stressful life events* will be assessed using the German-version of the Child and Adolescent Survey of Experiences (CASE-C/P [44]). The questionnaire is filled out by both the parent and child and consists of 38 pleasant or unpleasant events that the child may have experienced in the past 12 months. The CASE demonstrates good test-retest reliability (0.75) und good correlation with an interview-based measure of stressful life events [44].

The moderating role of *parental diagnostic status* (current or previous episode of depression) will be assessed using the DIPS [30] interview conducted at T1.

### Statistical analysis and estimated sample size

Intention-to-treat analyses are planned, such that all families who are randomised are included in the final analyses. We will use t-tests and chi-squared tests to check whether randomisation is successful. Interclass correlations (ICC) will be calculated in order to examine the extent to which values for the various outcome measures correlate between members of the same family. Confounding variables (demographics, parental depressive symptoms, course of parental therapy) will be included in statistical models.

In order to assess the impact of the intervention on the primary outcome (depression diagnosis) logistic regression models will be used to generate Odds Ratios (OR). Survival analysis will also be used to measure whether the time to a diagnosis of depression varies between the two groups [16]. In order to assess the clinical efficacy of the programme, the number of participants needed to treat (NNT) to prevent an incidence of depression will also be calculated.

The statistical analysis of the secondary outcome (child’s psychopathological symptoms) will be performed using multi-level regression, with group (intervention vs control) as the predictor variable.

We will also use regression models to assess the influence of moderating (stressful life events and parental diagnostic status) and mediating (knowledge of depression, coping strategies, parenting style, attributional style) variables.

### Sample size

We based our sample size calculation on effect sizes found in previous studies. Based on the results of a similar study which recruited the children of depressed parents and included a treatment as usual control group [16,17], we expected 33% of children in our control group to meet criteria for depression at 15 month follow-up (previous studies report 33% at 9 month follow-up [17] and 48% at 24 month follow-up [16]). Based on a study using the same prevention intervention [9,25], we expected around 10% of the experimental group to meet criteria for an episode of depression at follow-up (Compas and colleagues report 9% at 9 month follow-up [9] and 14% at 24 month follow-up [25]). A one-sided Fischer exact test comparing these proportions (10% vs 33%), with an alpha value of 5% and power of 80%, indicates a necessary sample size of 46 families in each group (N = 92 in total). Our aim is therefore to recruit 100 families, although it should be noted that our calculations are limited, since no previous study has recruited children of depressed parents with no previous psychiatric history themselves, or compared the intervention program we intend to use with a treatment as usual control condition.

## Discussion

Children whose parents have suffered from depression have an elevated risk of developing depression themselves, yet there is little formal support available to such children. An effective preventive intervention for this vulnerable group could convey significant personal, social, and economic gains. Although a promising preventive programme which includes both parents and children, and adopts principles of CBT, shows encouraging results, these are yet to be replicated [9]. This protocol describes an RCT to evaluate whether this intervention can be applied to a German sample to reduce the risk of depression in psychiatrically healthy children whose parents suffer from depression.

One hundred participating families will be randomly allocated to receive either i) the GGA preventive intervention, or ii) no intervention. The intervention is a 12-session group- and family-based cognitive-behavioural programme adapted here for the first time in German. The primary outcome measure will be diagnostic status (depression) at 15-month follow-up and the secondary outcome will be psychopathological symptoms immediately after the intervention, as well as at 9- and 15-month follow-up. We hypothesise that in comparison to usual care, the intervention will be effective in significantly reducing children’s risk of a depressive episode and psychopathological symptoms. We expect these improvements in the experimental group to be associated with improvements in knowledge of depression, the child’s coping strategies and their attributional style, and the parents’ parenting skills. We expect families who experience stressful life events during the course of the study, and families in which the parent is not currently depressed, to show the greatest benefits from the programme.

### Risks and side-effects

Existing studies of preventive interventions for families with a parent who is, or has, suffered from depression, provide no evidence of any associated risks or complications. Despite this low-risk, spontaneously reported side-effects of the intervention will be documented by the study leaders and will be discussed in regular supervision sessions. The detailed diagnostic interview conducted at the beginning of the study will ensure that no parents or children are included in the study who require a different type of treatment. Families are entitled to withdraw participation in the study at any point and do so with no disadvantage. During the study, if depression (or another psychiatric disorder) is recognised or suspected in the child, the child and parents will be informed and be offered the opportunity to receive treatment in the clinic for child and adolescent psychiatry. Parents who receive a diagnosis of depression for the first time during the study period will be encouraged to seek treatment at the clinic for psychiatry. Continued participation in the study is nevertheless possible.

## Abbreviations

ASF-KJ: Attribution style questionnaire for children and adolescents
BDI-II: Beck depression inventory
CASE-C/P: Child and adolescent survey of experiences
CBCL: Child behaviour checklist
CBT: Cognitive-behavioural therapy
CFT 20-R: Culture fair intelligence test
CWD: Coping with depression
DIKJ: Depression inventory for children and adolescents
DIPS: Diagnostic interview for psychiatric disorders
DSM-V: Diagnostic and statistical manual for mental disorders
ESI: Parenting style inventory
FEEL-KJ: Questionnaire for the assessment of emotion regulation in children and adolescents
FTI: Family talk intervention
GGA: Gesund und Glücklich Aufwachsen – *“Grow up healthy and happy”*)
ICC: Inter-class correlations
K-DIPS: Diagnostic interview for psychiatric disorders for children and adolescents
LMU: Ludwig-Maximilians University
NNT: Number needed to treat
RCT: Randomised controlled trial
SPIRIT: Standard protocol items: recommendations for interventional trials
YSR: Youth self-report
